# Special Issue “New Insights into Adipose Tissue Metabolic Function and Dysfunction, 3rd Edition”

**DOI:** 10.3390/ijms26167831

**Published:** 2025-08-13

**Authors:** Federica Mannino

**Affiliations:** Department of Medicine and Surgery, University of Enna “Kore”, Contrada Santa Panasia, 94100 Enna, Italy; federica.mannino@unikore.it

The understanding of adipose tissue has evolved from viewing it as a passive storage depot for excess energy to recognizing it as a central endocrine organ, crucial in regulating metabolic homeostasis, immune responses, and inter-organ communication. It secretes a wide array of bioactive molecules, adipokines, cytokines, and lipids that modulate systemic energy balance, insulin sensitivity, inflammation, and even behavior [1,2]. Dysfunctional adipose tissue, often resulting from a chronic positive energy balance, is a hallmark of obesity and is strongly implicated in the pathogenesis of insulin resistance, type 2 diabetes, atherosclerosis, and various cancers [3,4,5].

Obesity is not merely an increase in fat mass but reflects a profound alteration in adipose tissue structure and function, marked by hypoxia, fibrosis, macrophage infiltration, and a pro-inflammatory shift in secretory profile [6,7]. These changes disrupt lipid buffering, impair insulin signaling, and promote systemic metabolic dysfunction. Furthermore, adipose tissue plays a role in nutrient sensing, circadian biology, and even thermogenesis through beige and brown fat depots, whose functional decline may exacerbate metabolic disease [8,9].

The global obesity epidemic has amplified the need to understand the biology of adipose tissue at multiple levels, from genetics and epigenetics to microbiome interactions and lifestyle factors. Despite the progress, many questions remain regarding how adipose tissue adapts or maladapts under nutritional, hormonal, and environmental stressors.

This Special Issue of the *International Journal of Molecular Science*, titled "New Insights into Adipose Tissue Metabolic Function and Dysfunction, 3rd Edition", brings together a multidisciplinary collection of studies exploring these key questions. It includes investigations into developmental programming, genetic variants, dietary and physical activity interventions, molecular mechanisms of inflammation, and innovative tools such as metabolomics and systems biology.

The contributions reflect the field’s shift toward a more integrative understanding of adipose tissue, not just as a site of energy storage but as a complex and dynamic organ central to metabolic health and disease.

The concept of developmental programming, also known as the Developmental Origins of Health and Disease (DOHaD), proposes that environmental influences during critical periods of prenatal and early postnatal development can have lasting effects on an individual’s risk for obesity and metabolic disease [10]. Among the most potent of these influences is early-life nutrition, which shapes not only adiposity but also the function and structure of adipose tissue itself, as well as appetite regulation, insulin sensitivity, and immune responses [11,12]. In this context, maternal obesity and malnutrition have been strongly associated with an increased risk of metabolic dysfunction in offspring. These effects are thought to be mediated through epigenetic mechanisms, alterations in hypothalamic development, changes in adipose tissue precursor cells, and disruptions in gut microbiota [13,14]. For example, offspring exposed to maternal high-fat diets show increased adiposity, insulin resistance, and pro-inflammatory profiles even when raised on a normal diet post-weaning [15].

This Special Issue includes two contributions that provide compelling evidence for the long-term metabolic impact of early-life dietary exposures and potential preventive strategies. In a rodent model of maternal obesity, Pomar et al. [16] demonstrate that either dietary improvement during gestation or leptin supplementation during the suckling period attenuates the elevation of pro-inflammatory cytokines and oxidative stress markers in adult offspring. These findings reinforce leptin’s role not just as a satiety hormone but also as a neurotrophic and immunomodulatory factor critical during early postnatal development [17]. Complementing this, Bazhan et al. [18] reveal that the timing of exposure to a hypercaloric cafeteria diet significantly impacts the obesity phenotype in mice, and that these effects differ between sexes. Early-life exposure exacerbates weight gain and adipose tissue accumulation more profoundly in males than females, suggesting a sex-specific programming of energy metabolism and fat distribution, a phenomenon supported by earlier work in both animal and human studies [19].

A growing body of evidence indicates that genetic factors and molecular signaling pathways play a pivotal role in the development and progression of adipose tissue dysfunction. Monogenic and polygenic forms of obesity can result from variants affecting genes involved in appetite regulation, adipogenesis, insulin signaling, lipolysis, and mitochondrial function [20]. Disruption of these processes can impair adipose tissue expandability, alter its secretory profile, and promote chronic inflammation, ultimately contributing to systemic metabolic disease [21]. In this Special Issue, Mohammed et al. [22] present a functional characterization of a novel homozygous variant in the ADCY3 gene, which encodes adenylate cyclase 3, a key enzyme in cyclic AMP production implicated in hypothalamic signaling and energy homeostasis. Loss-of-function mutations in ADCY3 have been linked to early-onset severe obesity and impaired olfactory signaling, suggesting its dual role in both central and peripheral energy regulation [23,24]. The study highlights the relevance of gene sequencing and functional validation in uncovering pathogenic mechanisms of inherited obesity.

At the cellular level, chronic low-grade inflammation within adipose tissue is a hallmark of dysfunction and insulin resistance. In particular, advanced glycation end products (AGEs) have emerged as key modulators of adipocyte metabolism, acting through receptors such as RAGE and signaling intermediates including NF-κB. The study by Michalani et al. [25] shows that AGE exposure leads to downregulation of the Slc2a4 gene, which encodes the glucose transporter GLUT4, via NF-κB activation in 3T3-L1 adipocytes. This aligns with previous findings demonstrating that inflammatory pathways can impair insulin signaling at the transcriptional level, contributing to reduced glucose uptake and metabolic inflexibility [26,27]. More broadly, epigenetic mechanisms, such as DNA methylation, histone modifications, and microRNAs, are increasingly recognized as mediators of the effects of both genetic predisposition and environmental exposure on adipose tissue biology [28]. For example, obesity has been associated with methylation changes in loci related to adipogenesis and lipid metabolism, and such epigenetic marks may also serve as biomarkers for metabolic risk or targets for future therapies [29].

In the face of the growing global obesity epidemic, lifestyle-based interventions remain the cornerstone for the prevention and management of adipose tissue dysfunction. Exercise and dietary modulation not only reduce adiposity but also improve adipose tissue phenotype, enhance mitochondrial activity, and restore insulin sensitivity [30]. Importantly, emerging evidence suggests that the quality, timing, and composition of nutritional intake, as well as bioactive compounds from functional foods, can influence adipose tissue metabolism independently of weight loss [31]. Aerobic exercise, for example, has been shown to induce anti-inflammatory changes in adipose tissue, promoting a shift from pro-inflammatory M1 to anti-inflammatory M2 macrophage polarization, enhancing GLUT4 expression, and improving adipokine profiles [32]. In this Special Issue, Del Bianco et al. [33] report that aerobic training ameliorates insulin resistance and adipose tissue inflammation in rats, even in the context of a low-sodium diet, which paradoxically increases visceral adiposity and inflammatory markers. This study highlights the complexity of diet–exercise interactions, emphasizing that exercise can mitigate some of the unintended metabolic consequences of certain restrictive diets. Dietary interventions also play a crucial role. Erta et al. [34] examine the metabolic effects of a 12-week dietary intervention in overweight women of reproductive age, demonstrating improvements in key adipose tissue markers, including leptin and adiponectin levels. Their findings support previous clinical research showing that even modest dietary changes can significantly alter the endocrine and inflammatory function of adipose tissue, with implications for fertility, metabolic health, and long-term disease risk [35,36]. In parallel, the role of functional foods and bioactive compounds in modulating adipose tissue function is gaining attention. Hyun et al. [37] explore the metabolic potential of L-fucose-rich sulfated glycans derived from brown seaweed, showing that they enhance energy expenditure and mitigate obesity phenotypes in animal models. These findings are aligned with a growing body of literature showing that seaweed-derived polysaccharides, such as fucoidan and alginate, possess prebiotic, anti-inflammatory, and thermogenic properties that can beneficially influence adipose tissue and systemic metabolism [38,39]. Furthermore, dietary polyphenols, omega-3 fatty acids, and other phytochemicals have demonstrated the capacity to regulate adipocyte differentiation, stimulate the browning of white adipose tissue, and modulate oxidative stress pathways [40]. The integration of such functional food components into conventional dietary approaches represents a promising strategy to target adipose dysfunction without relying solely on caloric restriction.

Understanding the complex regulatory networks that govern adipose tissue function has become essential for identifying biomarkers predictive of obesity progression and its metabolic complications. Modern systems biology approaches, including metabolomics, microRNA profiling, and integrative omics, have revolutionized our capacity to detect subtle changes in adipose physiology that precede overt disease [41]. In this Special Issue, Skowronek et al. [42] conduct a scoping review on the potential of metabolomics in obesity research, highlighting how shifts in amino acid, lipid, and carbohydrate metabolites may reflect alterations in adipocyte function and systemic insulin sensitivity. Metabolomic profiles are particularly promising for uncovering early biomarkers of metabolic dysfunction, enabling stratification of obese individuals by risk and responsiveness to therapy [43]. For instance, elevated circulating levels of branched-chain amino acids (BCAAs) and certain acylcarnitines have been consistently associated with insulin resistance and visceral adiposity, likely reflecting impaired mitochondrial oxidative capacity in adipose and muscle tissue [44]. A second emerging layer of regulation is provided by non-coding RNAs, especially microRNAs (miRNAs), which fine-tune gene expression post-transcriptionally. Dysregulation of specific miRNAs, such as miR-143, miR-103, and miR-21, has been implicated in the promotion of adipogenesis, chronic inflammation, and insulin resistance [45]. In their systematic review, Azari et al. [46] underscore the functional interplay between gut microbiota and host miRNAs, suggesting that microbial-derived metabolites can influence host gene expression via miRNA modulation, creating a bidirectional regulatory axis between the gut and adipose tissue. This emerging field of microbiota and miRNA crosstalk may help explain the persistent effects of diet and environment on adipose tissue phenotype, even after weight normalization. Further contributing to the regulatory complexity is the incretin system, traditionally associated with glucose metabolism via pancreatic effects, but now recognized as a modulator of adipose tissue activity. In their thought-provoking perspective, De Fano et al. [47] propose adipose tissue as a novel target of the incretin axis, suggesting that GLP-1 and GIP receptors in adipocytes may influence lipolysis, browning, and cytokine secretion. This paradigm shift aligns with emerging data indicating that incretin-based therapies not only improve glycemia but also reduce fat mass and inflammation in adipose depots [48,49].

Once regarded as a passive energy depot, adipose tissue is now widely recognized as a highly dynamic endocrine organ, capable of secreting a wide array of hormones, cytokines, and lipid mediators that coordinate systemic metabolism. Among these, adipokines such as leptin, adiponectin, resistin, and visfatin have emerged as key players in energy homeostasis, glucose uptake, and inflammatory modulation [2,50]. The complexity of adipose-derived hormonal signaling is further amplified by its crosstalk with other endocrine systems, including the gut–brain axis, hypothalamic–pituitary–adrenal (HPA) axis, and incretin hormones. De Fano et al. [47]’s insight aligns with emerging evidence from GLP-1 receptor agonist studies, which show that incretin-based therapies not only reduce glycemia and appetite but also improve adipose tissue inflammation and insulin sensitivity, independently of weight loss [48,51]. Recent studies have also highlighted the bidirectional communication between adipose tissue and the hypothalamus, mediated by both hormonal and neural pathways. For example, leptin and adiponectin not only regulate hypothalamic appetite centers but are themselves modulated by neuroendocrine signals such as melanocortins and corticotropin-releasing hormone (CRH), creating feedback loops essential for metabolic adaptation [52]. Additionally, adipose tissue glucocorticoid metabolism, via 11β-HSD1 activity, serves as a local amplifier of HPA axis effects, contributing to visceral adiposity and insulin resistance [53]. Furthermore, recent discoveries have expanded the functional repertoire of adipose tissue to include exosomal and extracellular vesicle (EV) signaling, which allow adipocytes to influence distant tissues through the transport of miRNAs, proteins, and lipids. These vesicles have been shown to modulate insulin signaling in skeletal muscle, promote macrophage polarization, and even impact hepatic gluconeogenesis, establishing adipose tissue as a key inter-organ communication hub [54,55].

In conclusion, the studies included in this Special Issue reveal a highly integrated view of adipose tissue biology, ranging from molecular genetics and epigenetic regulation to dietary and hormonal interventions. They collectively highlight adipose tissue not only as a metabolic organ but also as a central node in immune–endocrine crosstalk and systemic homeostasis, serving as a valuable resource for researchers and clinicians working to unravel the complex mechanisms of adipose dysfunction and to develop innovative strategies for the prevention and treatment of metabolic disease.
